# Electroactive Composites with Block Copolymer-Templated Iron Oxide Nanoparticles for Magnetic Hyperthermia Application

**DOI:** 10.3390/polym11091430

**Published:** 2019-08-31

**Authors:** Shu-Chian Yang, Chun-Yu Chen, Hung-Yu Wan, Szu-Ying Huang, Ta-I Yang

**Affiliations:** Department of Chemical Engineering, Chung-Yuan Christian University, Taoyuan 330, Taiwan

**Keywords:** iron oxide nanoparticles, electroactive composites, magnetic hyperthermia, block copolymer

## Abstract

Cancer has been one of the leading causes of human death for centuries. Magnetic hyperthermia is a promising technique to confine and control cancers. However, particles used in magnetic hyperthermia leaking from where the cancers are located could compromise human health. Therefore, we developed electroactive iron oxide/block copolymer composites to tackle the leakage problem. Experimental results show that oleylamine-modified magnetic iron oxide (Fe_3_O_4_) particles and electroactive tetraaniline (TA) could be templated in the self-assembled microstructures of sulfonated [styrene-b-(ethylene-ran-butylene)-b-styrene] (S-SEBS) block copolymers. Various amounts of Fe_3_O_4_ particles and TA oligomer were incorporated in S-SEBS block copolymer and their electroactive behavior was confirmed by exhibiting two pairs of well-defined anodic and cathodic current peaks in cyclic voltammetry tests. The heating performance of the resultant TA/Fe_3_O_4_/polymer composites improved on increasing the added amount of Fe_3_O_4_ particles and TA oligomers. Both Fe_3_O_4_ and TA can contribute to improved heating performance, but Fe_3_O_4_ possesses a greater contribution than TA does. Hence, the main source for increasing the composites’ temperature is Neel relaxation loss from Fe_3_O_4_ magnetic particles.

## 1. Introduction

Cancer has a major impact on human society across the world and researchers are exploring new strategies to control and manage cancer. Magnetic hyperthermia therapy is one of the promising methods without any major side effects to treat cancers [1,2].

Magnetic particles generate heat when exposed to an alternating magnetic field (AMF) due to the hysteresis loss and relaxational losses. The hysteresis loss is caused by the orientation of the magnetic moments, in multiple domains particles align continuously with the direction of the AMF [3,4]. On the other hand, relaxational losses occurs mainly in particles with a single magnetic domain and are due to the realignment of particles’ magnetic moments or particles’ attempt to realign themselves with the AMF [3,5]. Therefore, the magnetic particles could be inserted in the tumor region and, thus, lead to the local heating of the tumor biological tissue by AMF to treat cancer resulting from the reduced heat tolerance of malignant cells as compared with that of healthy cells [2].

Iron oxide nanoparticles, including magnetite (Fe_3_O_4_) and maghemite (γ-Fe_2_O_3_), are commonly utilized for magnetic hyperthermia therapy. Other magnetic ferrite nanoparticles with a combined composition of zinc, nickel, cobalt, or manganese are also used [6]. Numerous experimental results reported in literature have demonstrated the success of magnetic hyperthermia therapy in killing various cancer cells using magnetic particles, and it is currently undergoing clinical trials [3]. Nevertheless, most of nanoparticles are considered toxic and could cause adverse effects on human health such as impaired mitochondrial function, inflammation, and DNA damage. Therefore, particles used in magnetic hyperthermia leaking from where cancers are located could compromise human health [7,8,9,10,11]. In addition, it is not possible to maintain the repeated treatment advantage of magnetic hyperthermia since the particles for heating the cancer cells could be lost due to leakage [7].

Researchers are continuing to explore solutions for overcoming the nanoparticle leakage problem. The most convenient one is to encapsulate particles in polymers so that they could remain in polymers without leakage [7,11,12,13,14,15]. The polymers that have been utilized are chitosan, polycaprolactone, and poly(vinyl alcohol) fibers [12,13,15]. In addition, temperature-sensitive hydrogels, such as poly(organophosphazene), Pluronic^®^, and copolymers of *N*-isopropylacrylamide and *N*-hydroxymethyl-acrylamide, were also used to encapsulate particles for treating cancers [11,14,16]. Unfortunately, magnetic particles encapsulated in polymer fibers or hydrogels are usually highly aggregated. The clustering behavior of magnetic nanoparticles can drastically change their collective magnetic properties, which in turn may influence their magnetic hyperthermia performance [17,18]. Furthermore, aggregated particles also make theoretical modeling complicated, hindering a clear understanding of how magnetic nanoparticles behave in an AMF [19]. Therefore, the major challenge in magnetic cancer therapy using magnetic nanoparticles involves developing particle/polymer composites with controllable particle distribution so that the heating performance is predictable and reproducible [18].

Block copolymers are a class of macromolecules consisting of two or more chemically distinct polymer entities that are connected by covalent bonds. Usually, these blocks are thermodynamically incompatible so that they tend to phase-separate to different morphologies including spheres, hexagonally arranged columns, a gyroid phase, and a lamellar phase [20]. Therefore, these ordered nanostructures could be utilized to incorporate nanoparticles and control particle distribution and orientation precisely, enabling the development of nanocomposites with improved properties including magnetic, mechanical, optical, electrical, or barrier properties [21]. In addition to particles, the nanostructures generated from block copolymers could be utilized for the creation of ordered arrays of protein or peptides, showing great promise for controlling cellular behavior [22].

In study, we demonstrate a new strategy that utilizing a block copolymer enables magnetic polymer composites to have controllable particle distribution, which can tackle the particle aggregation problem for magnetic cancer therapy. The nature of the self-assembled nanoscale morphology of the block copolymer enables the incorporation of particles uniformly dispersed within the polymer matrix. Sulfonated [styrene-b-(ethylene-ran-butylene)-b-styrene] (S-SEBS) block copolymers containing sulfonic acid groups (–SO_3_H) were selected to template magnetic Fe_3_O_4_ nanoparticles within their self-assembled microstructures. In addition, aniline oligomers have been utilized for advanced applications in supercapacitors, sensors, drug delivery, tissue engineering, and therapeutic neural regeneration owing to their unique electrical, chemical, and optical properties [23,24,25]. For example, the aniline tetramer was introduced in agarose–alginate hydrogels and, thus, improved the cell proliferation benefitting from its high conductivity promoting cell signaling [26]. Therefore, the electroactive tetraaniline (TA) was also introduced in S-SEBS block copolymers to study its contribution to cancer hyperthermia therapy.

## 2. Experimental

### 2.1. Materials

FeCl_3_·6H_2_O (99%, Showa, Tokyo, Japan), oleylamine (>50.0%, TCI, Tokyo, Japan), and N-phenyl-p-phenylenediamine (98%, Alfa Aesar, Ward Hill, MA, USA) were used as received without further purification. Sulfonated (styrene-b-(ethylene-ran-butylene)-b-styrene) (S-SEBS) block copolymer solution was purchased from Aldrich. The molecular weight of the S-SEBS block copolymer was 80,000 g/mol consisting of 29 wt.% styrene blocks and 55–65 mol.% of sulfonated styrene blocks. All chemicals were used as received.

### 2.2. Fe_3_O_4_ Nanoparticle Synthesis

The Fe_3_O_4_ nanoparticles were synthesized with the addition of the oleylamine surfactant by a hydrothermal method. FeCl_3_·6H_2_O (2 mmol), sodium hydroxide (4 mmol), and oleylamine (15 mmol) were mixed with 10 mL deionized (DI) water and 30 mL ethylene glycol. The resulting solution was placed in a closed container and reacted at 200 °C for 12 h. The resulting black precipitates were separated from the solution by using a magnet and then washed with tetrahydrofuran to remove solvents and unreacted precursors.

### 2.3. Synthesis of Tetraaniline (TA)

TA was synthesized according to the report in literature [27]. *N*-phenyl-p-phenylenediamine (2 g) was dissolved in a mixed solution of acetone (40 mL) and 1 N hydrochloric acid (HCl) (100 mL) and then its temperature was lowered to 0 °C. Subsequently, a solution of iron (III) chloride hexahydrate (4.9 g in 30 mL of 1 N HCl) was added into the resultant solution. The reaction was at 0 °C for 4 h. The product (emeraldine salt state of TA) was collected by centrifugation and washed with 1 N ammonium hydroxide solution, resulting in emeraldine base of TA.

### 2.4. S-SEBS Templated Fe_3_O_4_/TA Nanoparticles

Fe_3_O_4_/TA/S-SEBS nanocomposites were prepared using a solution-casting method. The S-SEBS solutions were mixed with required amounts of synthesized Fe_3_O_4_/TA particles. The solution was vigorously stirred for 30 min. A solid film was formed by static casting over a period of one week.

### 2.5. Electrochemical Cyclic Voltammetry (CV) Study of TA/S-SEBS Composites

The redox behavior of the prepared TA/S-SEBS composites was investigated using CV measurements. The TA/S-SEBS composite was cast on an indium tin oxide glass serving as a working electrode. The CV measurement was performed in 100 mL of 1.0 N hydrochloric acid solution. The testing potentials ranged from −0.2 to 1.0 V at a scan rate of 50 mV·s^−1^ using a silver/silver chloride reference electrode and a platinum counter electrode.

### 2.6. Heating Performance of Magnetically Induced Hyperthermia

The heating performance of the developed Fe_3_O_4_/TA/S-SEBS composites were studied by utilizing an external alternating current (AC) magnetic field produced by the coil of an induction heater (Power Cube 64/900, President Honor Industries Co., Ltd., Taiwan). The samples for testing were pre-heated at 37 °C and then subjected to an AC magnetic field of 94 kA/m and a frequency of 840 kHz. The temperature of the samples was recorded, and their heating performance was evaluated using the specific absorption rate (SAR) calculated from following expression [28]:

(1) $$SAR=\frac{\sum_{i}C_{pi}m_{i}}{m_{sample}}\frac{\Delta T}{\Delta t}|\begin{array}{c} t\to 0 \end{array},$$

where C_pi_ and m_i_ are specific heat capacity and mass for each component respectively (C_p_ = 0.69 J g^−1^K^−1^ for Fe_3_O_4_ particles, C_p_ = 1.75 J g^−1^K^−1^ for TA, and C_p_ = 1.3 J g^−1^K^−1^ for S-SEBS), and m_sample_ is the mass of the composite film for testing. ΔT/Δt is the initial slope of the time-dependent heating curve. The SAR was calculated by the initial temperature change (t→0) after turning on the AC magnetic field in order to minimize the interference of the energy exchange between the testing composite film and the surroundings.

## 3. Results and Discussion

### 3.1. Structural Characterization of 12 nm Fe_3_O_4_ Seeds

The challenge for iron oxide nanoparticle synthesis is how to control particle formation and thus obtain particles having desired sizes without any aggregation. Oleylamine (OAM) is a long-chain primary alkylamine, which has shown its capability as a solvent, surfactant, and reducing agent for synthesizing nanoparticles with desired morphology and composition [29]. Experimental results in literature reported that monodisperse, magnetic cobalt ferrite (CoFe_2_O_4_) and zinc ferrite (ZnFe_2_O_4_), and nickel ferrite (NiFe_2_O_4_) could be synthesized by using OAM as a stabilizing agent and solvent [30,31]. Therefore, we utilized OAM acting as a stabilizing agent to synthesize magnetic iron oxide nanoparticles with narrow size distribution.

**Figure 1:**
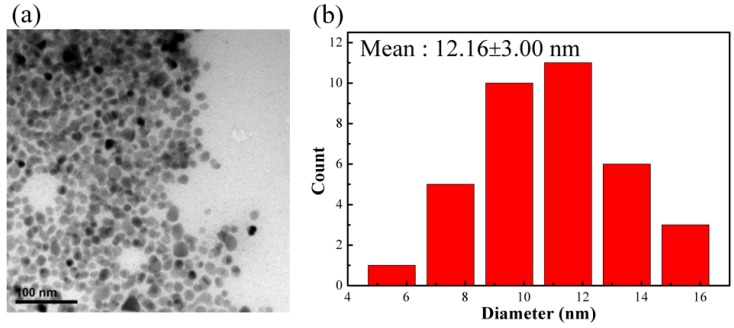
(**a**) TEM image of surfactant-modified Fe_3_O_4_ nanoparticles (scale bar = 100 nm); (**b**) particle size distribution.

The iron oxide nanoparticle synthesized by reacting 2 mmol FeCl_3_·6H_2_O in alkaline solution with the addition of 15 mmol oleylamine surfactant is nanoscale with narrow size distribution, as shown in Figure 1. The average size measured from TEM was 12.2 ± 3.0 nm. Furthermore, the obtained nanoparticles were highly soluble in organic solvents such as tetrahydrofuran or toluene. In addition, there was no aggregation between particles observed in TEM images. Evidence from FTIR test indicated the presence of OAM surfactant on the surface of synthesized particles as shown in Figure S1. Two sharp bands at 2923 and 2852 cm^−1^ are attributed to the –CH asymmetric stretching vibration and symmetric stretching vibration, respectively. In addition, the band at 1590, 1629, and 3008 cm^−1^ are characteristic of the –NH_2_, –C=C, and =C–H bending vibration, respectively [29]. These results confirm that OAM already modified the surface of iron oxide particles. In addition, TGA analysis revealed that the amount of OAM coated on the surface the particle is 15 wt.%, as shown in Figure S2. These results suggested that the surface of the nanoparticles were coated with OAM surfactant. The bulky hydrophobic part of the surfactant promoted nanoparticles soluble in nonpolar solvents and also provided the steric isolation needed to prevent particles from aggregation due to van der Waals attraction and magnetic attraction among magnetic particles.

**Figure 2:**
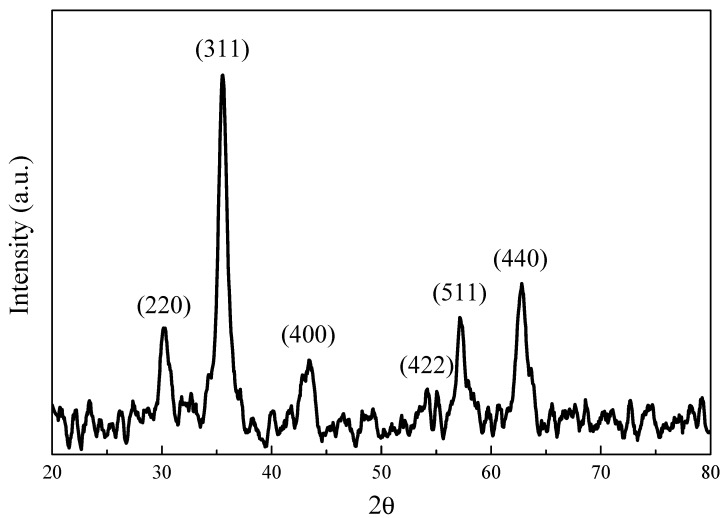
Wide-angle X-ray diffraction pattern of synthesized Fe_3_O_4_ nanoparticles.

**Figure 3:**
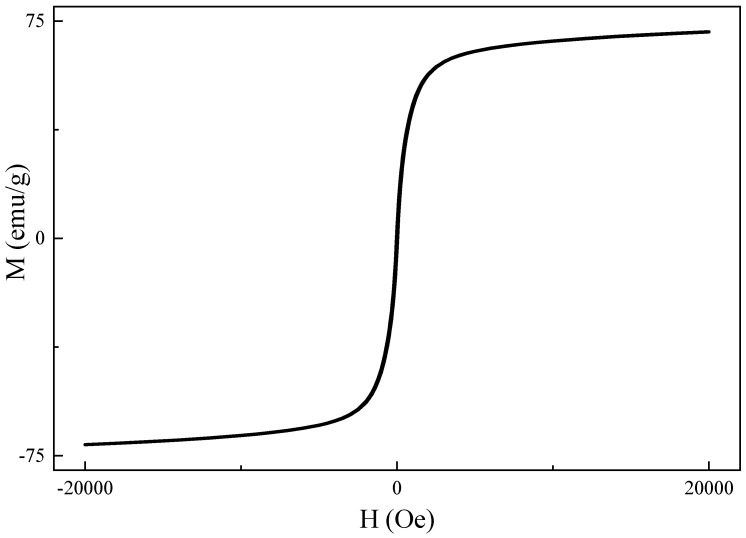
Magnetization (M) vs applied magnetic field (H) for surface-modified Fe_3_O_4_ nanoparticles at 300 K.

The chemical structure of the synthesized iron oxide was determined by XRD measurement (Figure 2). The diffraction peaks (2θ) of 30.35°, 35.95°, 43.45°, 53.70°, 57.25°, and 62.88° are consistent with X-ray diffraction from the (220), (311), (400), (422), (511), and (440) planes of face-centered cubic Fe_3_O_4_ (JCPDS 87-2334), indicating the synthesized iron oxide nanoparticles are magnetic Fe_3_O_4_ [32]. The magnetic properties of the synthesized 12 nm Fe_3_O_4_ nanoparticles were studied by SQUID (superconducting quantum interference device) at 300 K. The results (shown in Figure 3) revealed that there was no magnetization hysteresis observed as the applied magnetic field varied, indicating the magnetic particles are in a superparamagnetic state [33]. The particle size of the synthesized Fe_3_O_4_ nanoparticles is 12 nm, which is smaller than the 25 nm critical size for ferrimagnetic to superparamagnetic transition. Therefore, the synthesized Fe_3_O_4_ nanoparticles do not possess any magnetization when there is no applied external magnetic field [33]. Furthermore, their apparent saturation magnetization (Ms) is 71.2 emu/g. However, the actual Ms for the synthesized particles is 83.7 emu/g since there is 15 wt.% of OAM coated on their surface. However, it is still lower than the bulk value of Fe_3_O_4_ (90 emu/g) due to spin disorder arising as reported in literature [34]. Nevertheless, the synthesized Fe_3_O_4_ nanoparticles with superparamagnetic behavior are suitable for cancer hyperthermia therapy because their magnetization can be induced by an external magnetic field and no magnetization remains when it is removed.

### 3.2. Characterization of Electroactive Tetraaniline (TA)

**Figure 4:**
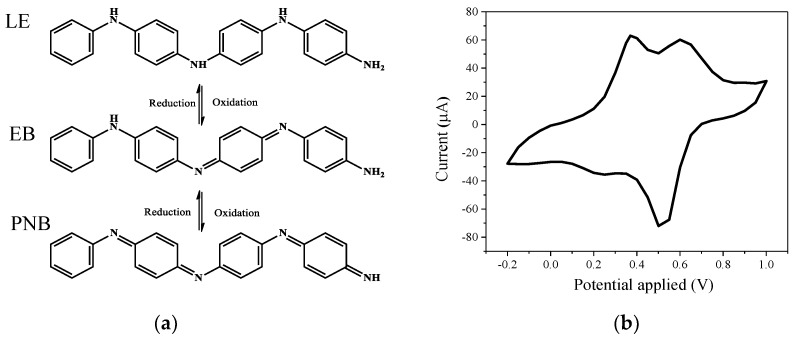
(**a**) Molecular structures of tetraaniline (TA) with different redox states, (**b**) cyclic voltammetry measurement for synthesized TA. LE—leucoemeraldine base; EB—emeraldine base; PNB— pernigraniline base.

There are three distinguishable oxidation states in TA, which are fully reduced (leucoemeraldine base) (LE), the half oxidized (emeraldine base) (EB), and the fully oxidized (pernigraniline base) (PNB) states as illustrated in Figure 4a. The synthesized TA was analyzed by FTIR (Figure S3). The peak of 1507 cm^−1^ could be assigned to benzenoid ring stretching vibrations [27]. The three peaks at 1383, 1151, and 864 cm^−1^ are from the C–N stretching vibration of a secondary aromatic amine, the aromatic C–H in-plane bending modes, and the C–H out-of-plane bending vibrations of 1,4-aromatic substituted benzene rings, respectively [27]. Moreover, a relatively strong peak close to 1671 cm^−1^ and the low intensity ratio of 1599 to 1510 cm^−1^ indicate the presence of a doped emeraldine salt (ES) state [27,35]. The CV tests for the synthesized TA in Figure 4b show that there are two oxidation peaks at 0.4 and 0.6 V vs. Ag/AgCl, which are attributed to the transition from fully reduced LE state to half oxidized EB state and half oxidized EB state to fully oxidized PNB state, respectively [23,36,37].

### 3.3. Electroactive Composites with Block Copolymer-Templated Iron Oxide Nanoparticles

**Figure 5:**
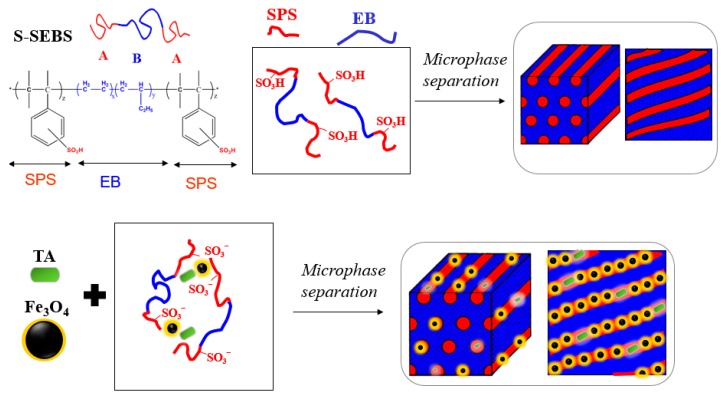
Templating TA and Fe_3_O_4_ nanoparticles within sulfonated (styrene-b-(ethylene-ran-butylene)-b-styrene) (S-SEBS) self-assembled microstructure.

We utilized a sulfonated (styrene-b-(ethylene-ran-butylene)-b-styrene) (S-SEBS) ABA-type triblock copolymer to template TA and Fe_3_O_4_ nanoparticles within its self-assembled microstructures, as illustrated in Figure 5, to develop electroactive, magnetic composites for cancer hyperthermia therapy.

Typical ABA-type triblock copolymer with 30 mol.% A block is expected to show hexagonal packed cylinder (HPC) morphology after phase separation. However, the S-SEBS polymer utilized in this study consisting of 29 wt.% styrene blocks and 55–65 mol.% of styrene blocks sulfonated. Therefore, the sulfonation of styrene blocks can disrupt the usual phase separated morphology so that the HPC mode is no longer present and, then, a worm-like morphology, which might be termed ‘frustrated’ owing to the comparative disorder, appears [38].

**Figure 6:**
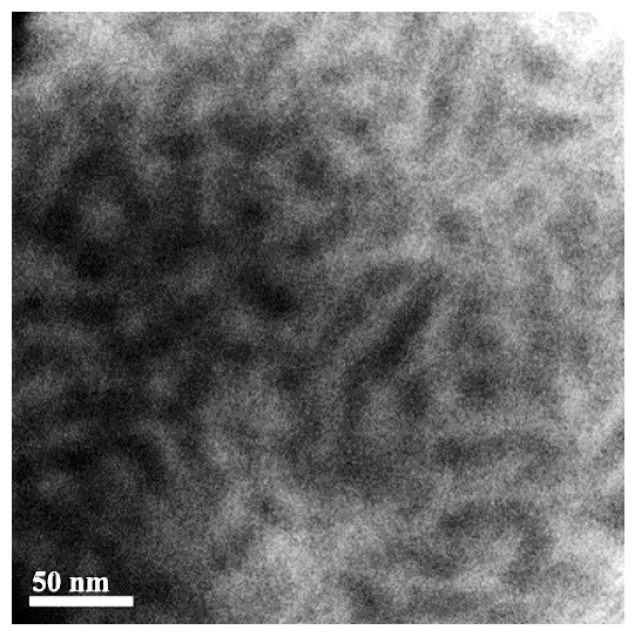
TEM image of S-SEBS block copolymer.

TEM image of our prepared S-SEBS polymer in Figure 6 exhibited stripe pattern, indicating formation of worm-like morphology. The darker stripes are the sulfonated styrene block (SSB) of S-SEBS block copolymers because TEM contrast originates from heavier chemical compositions, and, thus, the self-assembled SSB domain size is close to 20 nm determined from the TEM image. This atypical phase separation behavior contributes to the aggregation of the –SO_3_H ionic groups within the ionomeric blocks and the mixed solvents for dissolving the S-SEBS copolymer influence in the self-assembly process of the block microdomains during the casting process [39,40].

**Figure 7:**
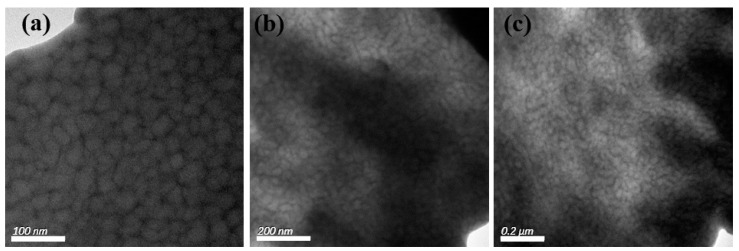
TEM image of S-SEBS polymer with various amounts of TA. (**a**) 1 wt.%; (**b**) 10 wt.%; (**c**) 20 wt.%.

**Figure 8:**
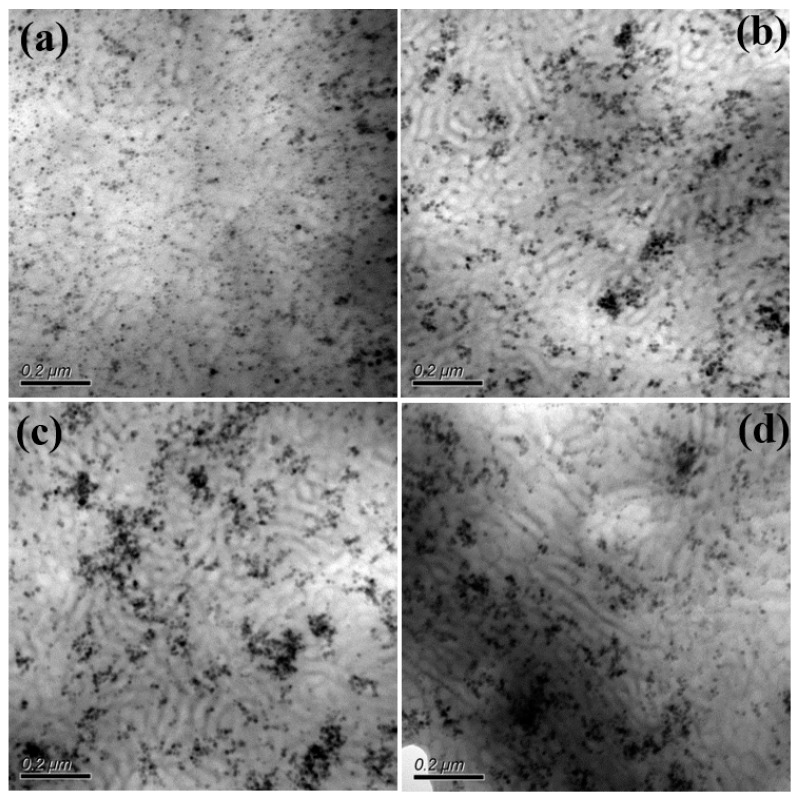
TEM image of S-SEBS polymer with various amounts of TA and Fe_3_O_4_. (**a**) 5 wt.% Fe_3_O_4_ and 15 wt.% TA (**b**) 10 wt.% Fe_3_O_4_ and 10 wt.% TA; (**c**) 15 wt.% Fe_3_O_4_ and 5 wt.% TA; (**d**) 20 wt.% Fe_3_O_4_.

For the S-SEBS block copolymer mixed with up to 20 wt.% electroactive TAs, the resulting microphase separated morphology was still “frustrated” worm-like morphology (Figure 7) and TAs were confined within the darker stripes containing the sulfonated styrene blocks due to π–π interactions. Furthermore, the synthesized Fe_3_O_4_ nanoparticles were also introduced in S-SEBS polymer to promote the heating performance of electroactive S-SEBS composites. The synthesized magnetic particles in this study were of 12 nm so that they could be easily incorporated in the block copolymer phase separation domain. The TEM image in Figure 8a shows that the 5 wt.% Fe_3_O_4_ nanoparticles with 15 wt.% TAs were successfully templated within the SSB microstructures of the S-SEBS copolymer. The sulfonic acid groups (–SO_3_H) can preferentially associate with OAM surfactant due to acid–base interactions and, thus, template the OAM-modified Fe_3_O_4_ nanoparticles within SSB microstructures of the S-SEBS polymer. However, some Fe_3_O_4_ nanoparticles aggregated together and could not be templated in the SSB domain when increasing the added amount of Fe_3_O_4_ nanoparticles to 10, 15, or 20 wt.% and maintaining the combined TA and Fe_3_O_4_ added amount at 20 wt.% (Figure 8b–d). These results contributed to the strong magnetic interaction between Fe_3_O_4_ nanoparticles so that they tended to aggregate and, thus, prevented them from being templated in the 20 nm SSB microstructure of the S-SEBS polymer. Nevertheless, most TA and Fe_3_O_4_ nanoparticles could be templated in the SSB microstructure of the S-SEBS polymers without showing severe aggregation.

### 3.4. Electrochemical Properties of Electroactive Fe_3_O_4_ Composites

**Figure 9:**
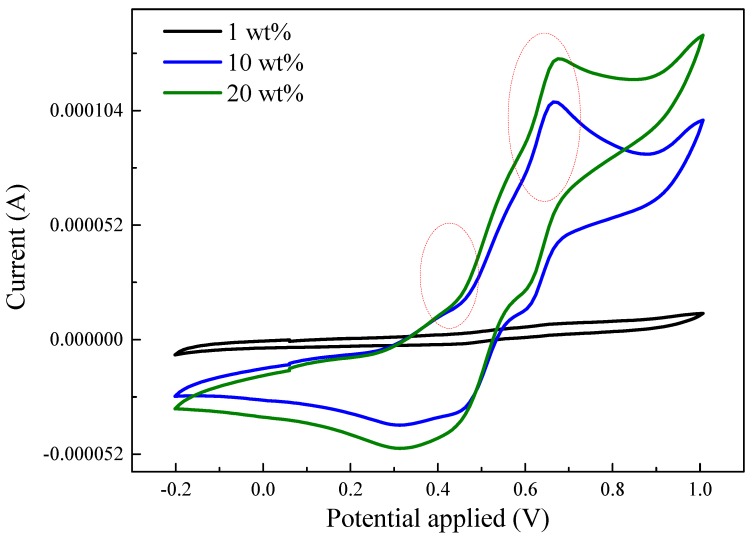
Cyclic voltammetry measurement for S-SEBS polymer with various amounts of TA.

The electrochemical-responsive behavior of TA/Fe_3_O_4_ composites was investigated in HCl solution. There are no distinct redox peaks present in the cyclic voltammetry test for the composite with only 1 wt.% TA as shown in Figure 9. However, the TA composites exhibits two pairs of redox peaks as the added amount increased to 10 wt.%. The first pair of redox peaks around the 0.4 V oxidation peak was due to the transition from the leucoemeraldine state to the emeraldine state. The second pair of redox peaks with the oxidation potential around 0.65 V was attributed to the transition from the emeraldine state to the pernigraniline state. Furthermore, the redox current increased on increasing the TA added amount to 20 wt.%. These results confirm that TA could enable the composites to have electroactive properties.

**Figure 10:**
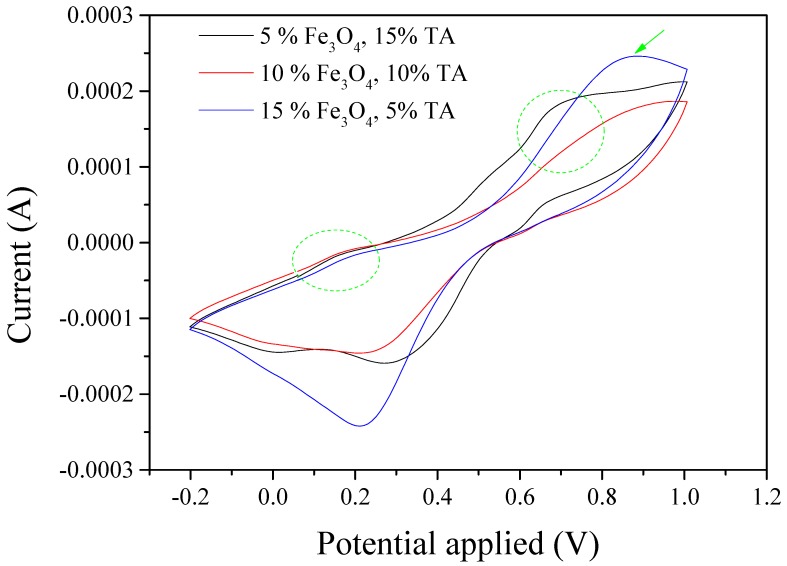
Cyclic voltammetry measurement for S-SEBS polymer with various amounts of TA and Fe_3_O_4_ nanoparticles. (Note: all % in the figure is wt.%.)

The CV test of the composite with 15 wt.% TA and 5 wt.% Fe_3_O_4_ shows that there are one weak and one strong peak around 0.2 and 0.65 V, respectively (Figure 10). This result suggests that the addition of Fe_3_O_4_ significantly influences the transition from the TA’s leucoemeraldine state to the emeraldine state compared to the transition from the emeraldine state to the pernigraniline state. The Fe_3_O_4_ began to affect TA’s transition from the emeraldine state to the pernigraniline state as its expected peak significantly diminished when the Fe_3_O_4_ increased to 10 wt.% and TA decreased to 10 wt.%. Moreover, it seems only one pair of redox peaks close to 0.8 and 0.2 V remained when the Fe_3_O_4_ amount continually increased to 15 wt.% and TA decreased to 5 wt.%. The oxidation peak and reduction peak for pure Fe_3_O_4_ are 0.8 and 0.2 V, respectively, as shown in Figure S4. Therefore, this result indicated that Fe_3_O_4_ dominated the CV results for the composite with 5 wt.% TA and 15 wt.% Fe_3_O_4_.

### 3.5. Hyperthermia Tests for Electroactive Fe_3_O_4_ Composites

This study focused on developing electroactive composites with controllable Fe_3_O_4_ particle distribution, which can tackle the particle aggregation problem for magnetic cancer therapy. We have shown the feasibility of utilizing a block copolymer to template electroactive TA and Fe_3_O_4_ nanoparticles in its self-assembled microstructure without showing severe aggregation. In order to reveal the potential of the developed TA/Fe_3_O_4_/polymer composites in hyperthermia application, their heating performance was evaluated by an alternating magnetic field and, thus, quantified using the specific absorption rate (SAR). A higher SAR value means better capability to provide heating power to increase the surroundings’ temperature.

**Figure 11:**
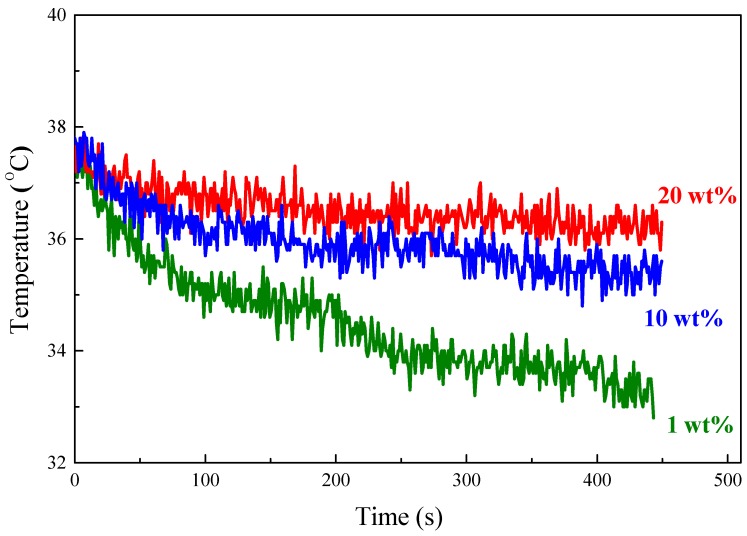
Heating performance of S-SEBS polymer with various amounts of TA.

TA has shown appreciable electrical conductivity for various applications [41]. Therefore, the temperature of the TA polymer composites is expected to increase resulting from the power dissipation from eddy currents, which is proportional to the electrical conductivity of materials, when subjecting to an AC magnetic field. The polymer composites with various amounts of TA for testing were pre-heated at 37 °C and then subjected to an AC magnetic field. The temperature of the samples was recorded immediately after turning on the AC magnetic field and their time-dependent heating curves are shown in Figure 11. The temperature for all three composites (1, 10, and 20 wt.%) increased at the beginning but decreased immediately because thermal energy flows from the composites to lower-temperature surroundings. Nevertheless, the composite with 20 wt.% TA showed the lowest temperature decrease rate compared to 1 and 10 wt.% TAs. These results confirm that the TA could be utilized as a heat source to release heat to the composite so that more TA in composites exhibited a lower temperature decrease rate.

**Figure 12:**
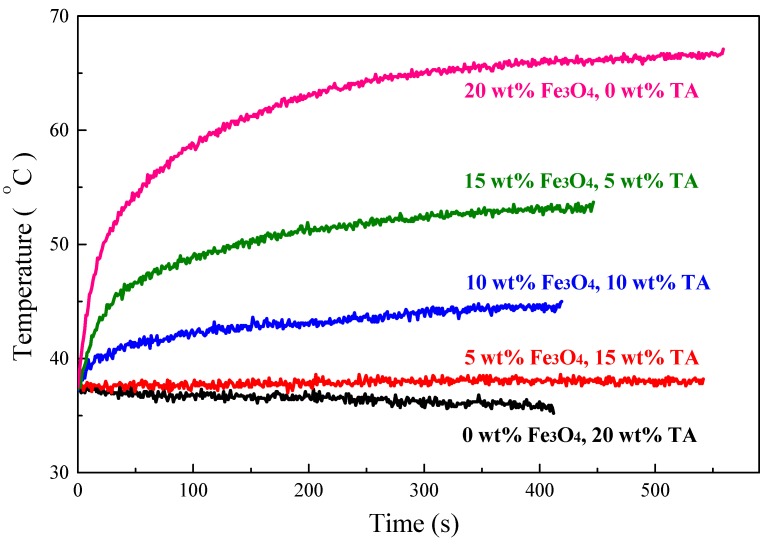
Heating performance of S-SEBS polymer with various amounts of TA and Fe_3_O_4_ nanoparticles.

In order to obtain composites with higher value of SAR, magnetic Fe_3_O_4_ nanoparticles combined with TA were introduced into S-SEBS polymer. TA could release heat due to eddy current loss when exposed to an alternating magnetic field (AMF). In contrast, the synthesized superparamagnetic Fe_3_O_4_ nanoparticles generate heat resulting from the Neel relaxation loss due to the rotation of the magnetic moment of the particles [4]. Figure 12 shows that adding Fe_3_O_4_ nanoparticles improved the heating performance of the composites. The temperature of the composite with 15 wt.% TA and 5 wt.% Fe_3_O_4_ particles increased, compared to the continuous temperature decrease of the composite only with 20 wt.% TA, and its temperature maintained at 38 °C, meaning that the heat generated by the composite is equal to the heat escaped to the surroundings. Furthermore, the heating temperature increased with increasing time and, then, reached equilibrium temperature at 44, 53, and 66 °C, respectively, when the Fe_3_O_4_ amount was increased to 10, 15, and 20 wt.%, while maintaining the combined TA and Fe_3_O_4_ added amount at 20 wt.%. The heating performance represented as specific absorption rate (SAR) for the TA/Fe_3_O_4_/polymer composites increased on increasing the added Fe_3_O_4_ amount as summarized in Table 1. These results suggest that the Neel relaxation loss from Fe_3_O_4_ nanoparticles outweighs the eddy current loss from TA and thus dominates the heating performance of TA/Fe_3_O_4_/polymer composites.

In summary, the heating performance of electroactive TA/Fe_3_O_4_/polymer composites increases on increasing the added amount of Fe_3_O_4_ particles and TA oligomers. Both Fe_3_O_4_ and TA can contribute to improved heating performance, but Fe_3_O_4_ possesses a greater contribution than TA does.

## 4. Conclusions

We have demonstrated the success of fabricating electroactive composites with block copolymer-templated iron oxide nanoparticles and TA oligomers for magnetic hyperthermia application. Magnetic Fe_3_O_4_ nanoparticles with uniform size distribution were synthesized by using an oleylamine surfactant acting as a stabilizing agent. The developed Fe_3_O_4_ nanoparticles with superparamagnetic behavior are suitable for cancer hyperthermia therapy because their magnetization can be induced by an external magnetic field and no magnetization remains when it is removed. Moreover, we utilized a sulfonated (styrene-b-(ethylene-ran-butylene)-b-styrene) (S-SEBS) ABA-type triblock copolymer to template electroactive TA and Fe_3_O_4_ nanoparticles within its self-assembled microstructures to develop electroactive, magnetic composites. Most TA and Fe_3_O_4_ nanoparticles could be templated in the styrene microstructure of the S-SEBS polymers without showing severe aggregation. The CV test revealed that the resultant composites possessed two distinct TA structure transitions from the leucoemeraldine state to the emeraldine state and from the emeraldine state to the pernigraniline state, respectively. Their heating efficiency was evaluated by an AC magnetic field. The results conclude that the heating performance of the resultant TA/Fe_3_O_4_/polymer composites increases on increasing the added amount of Fe_3_O_4_ particles and TA oligomers. Both Fe_3_O_4_ and TA can contribute to improved heating performance, but Fe_3_O_4_ possesses a greater contribution than TA does.

## Figures and Tables

**Table 1:** Specific absorption rate (SAR) values for S-SEBS polymer with various amounts of TA and Fe_3_O_4_ nanoparticles.

Composites	SAR (W/g)
0 wt.% Fe_3_O_4_ + 20 wt.% TA	−0.005
5 wt.% Fe_3_O_4_ + 15 wt.% TA	0.001
10 wt.% Fe_3_O_4_ + 10 wt.% TA	0.339
15 wt.% Fe_3_O_4_+ 5 wt.% TA	0.750
20 wt.% Fe_3_O_4_+ 0 wt.% TA	1.093

## Supplementary Materials

The following are available online at https://www.mdpi.com/2073-4360/11/9/1430/s1, Figure S1: FTIR spectroscopy spectrum of surface-modified Fe_3_O_4_ nanoparticles; Figure S2: Thermogravimetric analysis (TGA) of surface-modified Fe3O4 nanoparticles.; Figure S3: FTIR of synthesized TA; Figure S4: Cyclic voltammetry measurement for S-SEBS polymer with 20 wt.% Fe_3_O_4_ nanoparticles.
