# Supplementary material for: Electroactive Composites with Block Copolymer-Templated Iron Oxide Nanoparticles for Magnetic Hyperthermia Application
Source: Polymers (Basel). 2019 Aug 31;11(9):1430. doi: 10.3390/polym11091430 (PMC6780777; doi:10.3390/polym11091430)
Supplement: Supplementary file 1 [file polymers-11-01430-s001.pdf]

Supplementary materials

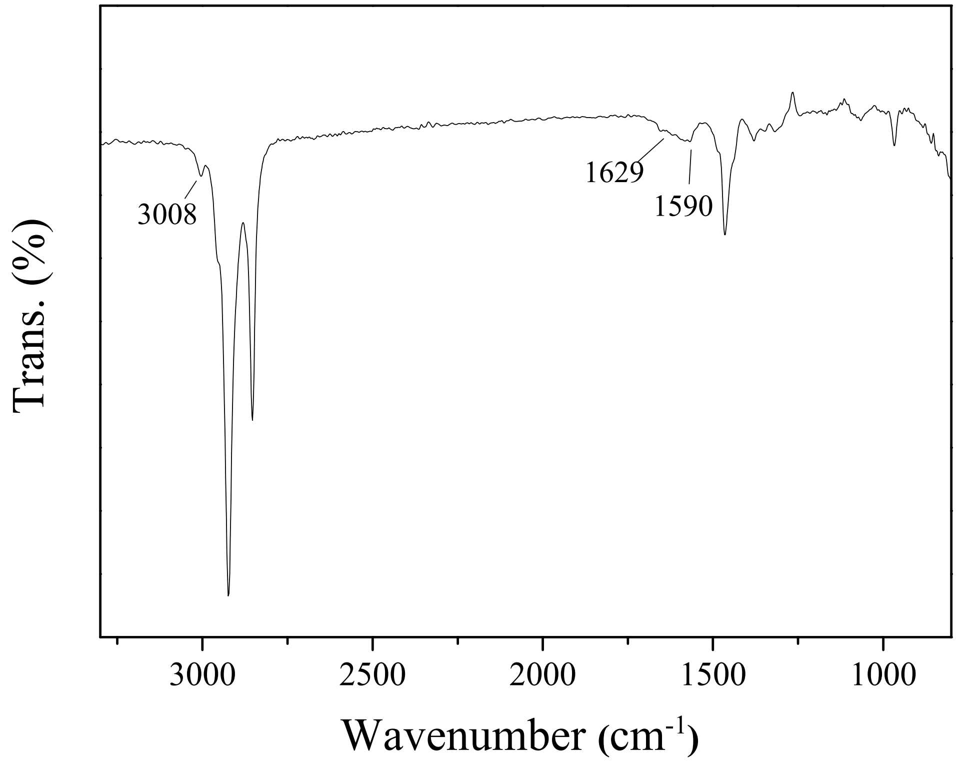

Figure S1. FTIR spectroscopy spectrum of surface-modified Fe_3O_4 nanoparticles.

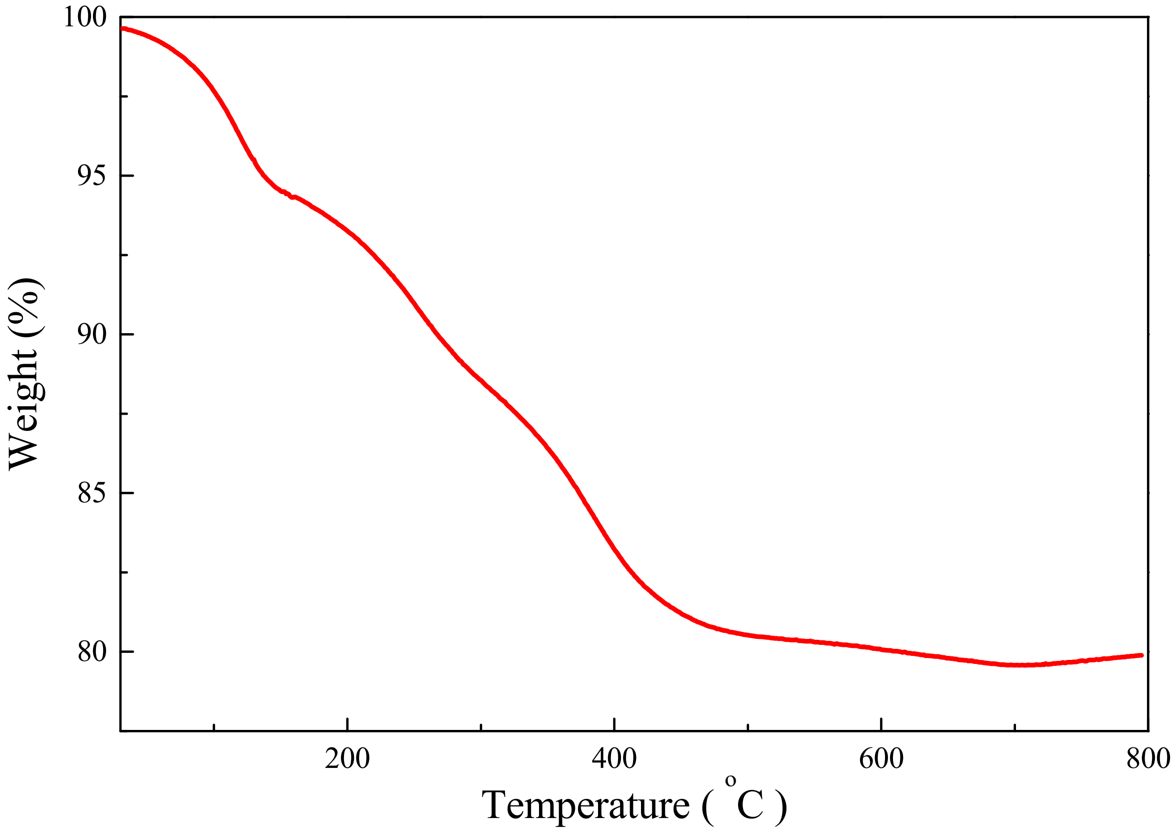

Figure S2. Thermogravimetric analysis (TGA) of surface-modified Fe_3O_4 nanoparticles..

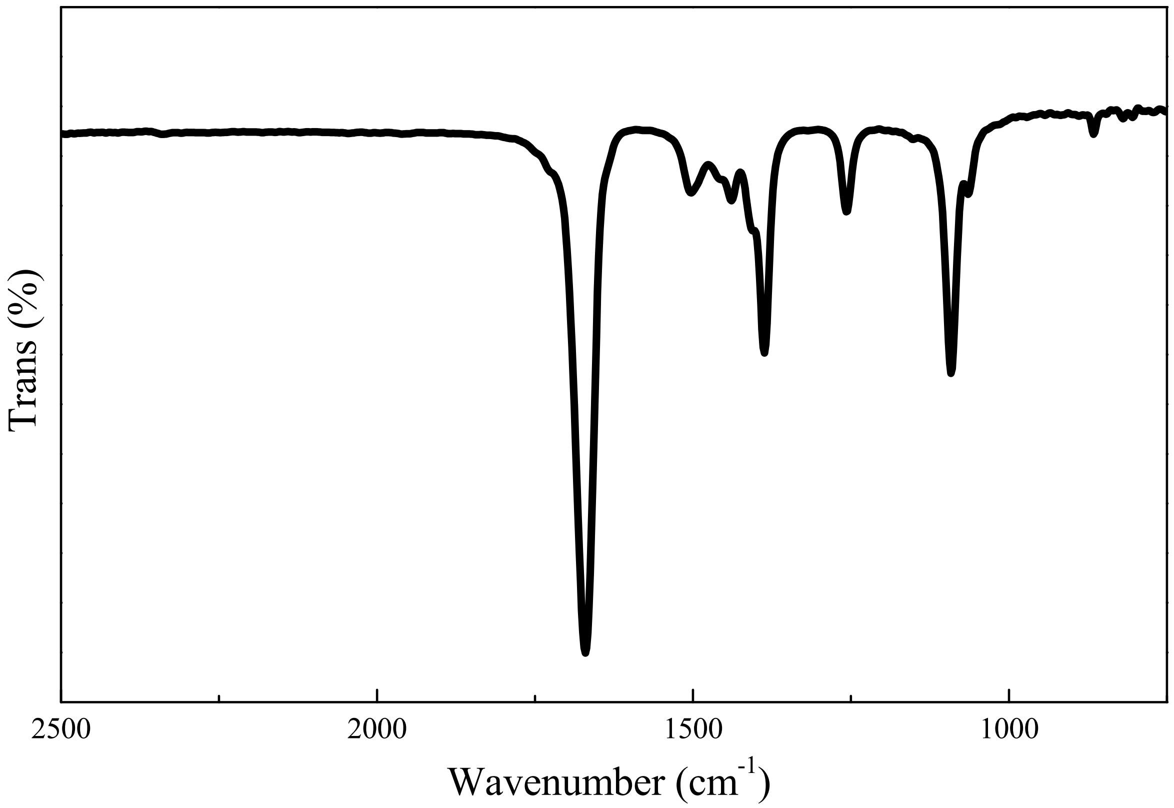

Figure S3. FTIR of synthesized TA.

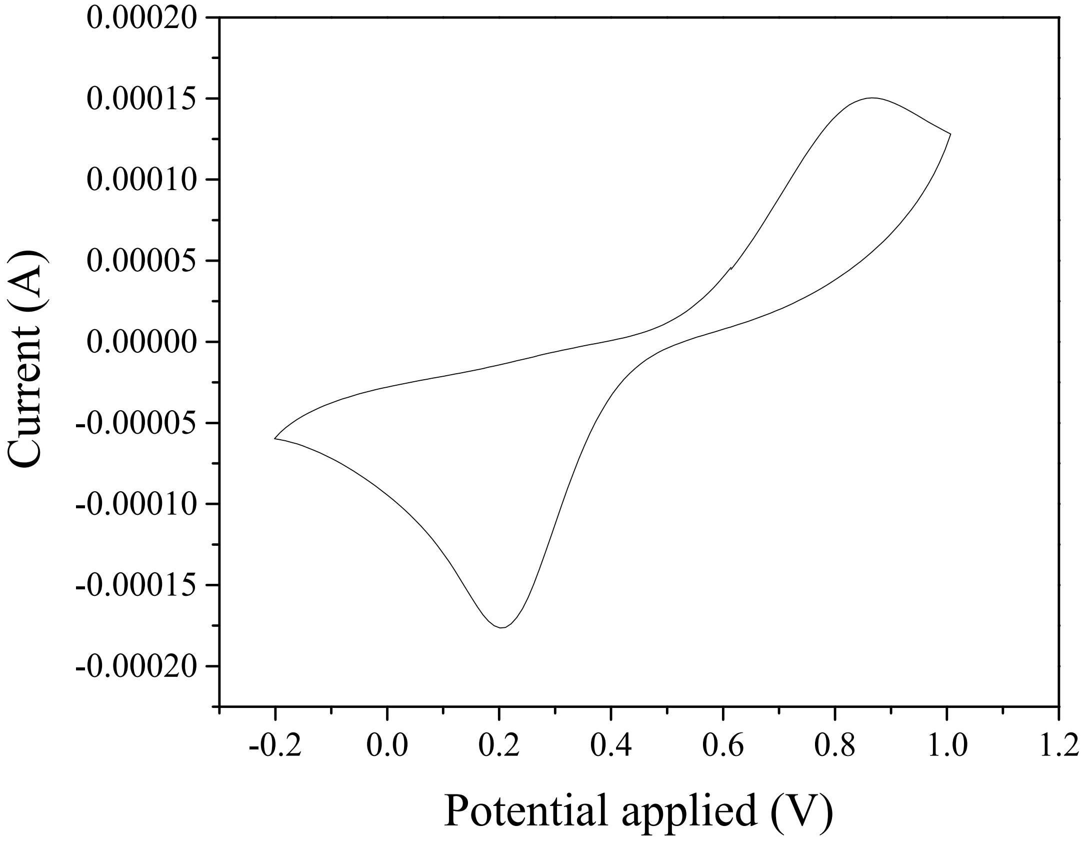

Figure S4. Cyclic voltammetry measurement for S-SEBS polymer with 20 wt% Fe_3O_4 nanoparticles.
